# Ludovico’s Technique: The Literary Depiction of Aversion Therapy in ‘A Clockwork Orange’

**DOI:** 10.1192/j.eurpsy.2022.2282

**Published:** 2022-09-01

**Authors:** J. Wellington, A. Wellington, M. Abbasy, M. Bhagia, D. Myles, V. Bhure

**Affiliations:** 1 Cardiff University, School Of Medicine, Barry, United Kingdom; 2 Cardiff University, Department Of Biosciences, Barry, United Kingdom; 3 Rush University Medical Centre, Medical Centre, Chicago, United States of America; 4 Nanavati Max Superspeciality Hospital, Medicine, Mumbai, India; 5 Medical University of the Americas, Psychiatry, Kentville, Canada; 6 Datta Meghe Institute of Medical Sciences, Institute Of Medical Sciences, Bhandara, India

**Keywords:** aversive conditioning, ludovico’s technique, Psychiatry in literature, Aversion therapy

## Abstract

**Introduction:**

Anthony Burgess’ novel ‘Clockwork Orange’ identifies the topical debates surrounding the use of aversion therapy (or aversive conditioning) as an effective treatment for addictive behaviours. Widely popularised in literature as ‘Ludovico’s Technique’, Burgess attempts to credit the misunderstanding and dramatization of its effects when the main protagonist is released from a prison sentence after undergoing this treatment.

**Objectives:**

We aimed to highlight the depictions of aversion therapy in modern popular literature.

**Methods:**

A narrative review of the current literature concerning aversion therapy and Anthony Burgess’s novel ‘A Clockwork Orange’ was conducted. Emphasis on the misinterpretation of aversive therapies was noted.

**Results:**

Since the introduction of pharmacological alternatives and additional forms of psychological therapies, there has been a decline in the use of aversion therapy in recent decades. However, it is still effective when conceding the conditioning process. Likewise, its predecessor’ visual imagery’ is believed to be a more acceptable and effective form.

**Conclusions:**

The depiction of aversion therapy in literature and media has played a role in shaping societal views on aversive conditioning techniques and the degree to which they are deemed acceptable forms of treatment. The “Ludovico Technique” featured in the novel ‘A Clockwork Orange’ and its film adaptation is arguably the most salient depiction of aversion therapy in popular culture.

**Disclosure:**

No significant relationships.

